# Noninversion Variants in Sporadic Hemophilia A Originate Mostly from Females

**DOI:** 10.3390/ijms26030891

**Published:** 2025-01-22

**Authors:** Ming Chen, Ming-Ching Shen, Shun-Ping Chang, Gwo-Chin Ma, Dong-Jay Lee, Adeline Yan

**Affiliations:** 1 Department of Genomic Medicine, Changhua Christian Hospital, Changhua 500, Taiwan; 104060@cch.org.tw (M.C.); 70914@cch.org.tw (S.-P.C.); 128729@cch.org.tw (G.-C.M.); 118862@cch.org.tw (D.-J.L.); 181435@cch.org.tw (A.Y.); 2 Department of Obstetrics and Gynecology, Changhua Christian Hospital, Changhua 500, Taiwan; 3 Department of Medical Genetics, National Taiwan University Hospital, Taipei 100, Taiwan; 4 Department of Obstetrics and Gynecology, National Taiwan University Hospital, Taipei 100, Taiwan; 5 Hemophilia Treatment and Thrombosis Center, Department of Internal Medicine, Changhua Christian Hospital, Changhua 500, Taiwan; 6 Department of Internal Medicine, National Taiwan University Hospital, Taipei 100, Taiwan; 7 Department of Laboratory Medicine, National Taiwan University Hospital, Taipei 100, Taiwan

**Keywords:** noninversion variant, sporadic hemophilia A, hemophilia A, de novo variant, female, mosaic variant, *F8* gene

## Abstract

*F8* gene inversion variants originate in male germ cells during spermatogenesis. Our recent study revealed that de novo variants (DNVs) caused *F8* noninversion variants (NIVs) in sporadic hemophilia A (HA). Here, we conducted a direct clinical determination of sex differences in the origin of sporadic HA-NIV according to the data of two new HA-NIV families, as well as of the families demonstrated in the previous study. Of the 126 registered families with HA, 23 were eligible for inclusion. We conducted a linkage analysis with *F8* gene markers and an amplification refractory mutation system–quantitative polymerase chain reaction to confirm the origin of the sporadic NIVs (~0% mutant cells) or the presence of a mosaic variant, requiring further confirmation of the origin in the parent. Two sporadic DNV events were determined. One event occurred in grandparents, consisting of five maternal grandmothers and seven maternal grandfathers, who were the origins; their respective daughters became carrier mothers who gave birth to probands. The other event included 11 mothers, who were the origins exclusively; their respective sons became probands. In this study, we found that sporadic HA-NIVs originate mostly from females (16 out of 23). Our study contributes to a better understanding of the genetic pathogenesis of HA.

## 1. Introduction

Hemophilia A (HA) is the most prevalent X-linked congenital bleeding disorder. It is caused by variants in the *F8* gene, resulting in deficiency or dysfunction of the coagulation protein, factor VIII (FVIII). The *F8* gene is located on chromosome Xq28, consisting of 26 exons and 25 introns, and encoding the FVIII protein comprised of 2332 amino acids [1]. *F8* gene variants are classified as inversion variants, consisting of intron 22 and intron 1 inversions or noninversion variants (NIVs), including point variants, deletions, insertions, or duplications [2,3,4]. FVIII functions as a cofactor for factor X activation by activated factor IX [5,6]. HA is clinically defined based on blood FVIII activity as either mild (>5–40 IU/dL), moderate (1–5 IU/dL), or severe (<1 IU/dL) [7]. The reliable estimates of the prevalence of HA, based on the World Federation of Hemophilia (WFH) annual global summary and national patient registry data, have shown that the estimated prevalence of HA at birth is 24.6 cases per 100,000 males for all severities of HA and 9.5 cases for severe HA [7].

Sporadic hemophilia involves the first affected patient with hemophilia determined in a family where no previous relative has hemophilia or has been proven to be a hemophilia carrier [8].

Sporadic hemophilia has enabled the persistence of hemophilia in the population for thousands of years [8,9]. We are interested in understanding how sporadic HA occurs. As *F8* gene inversion has been well studied [10,11], we are limiting our investigation to cases of sporadic NIVs over two or three generations of HA families. Our recent study [12] has revealed the following points: (a) de novo variants (DNVs) primarily caused *F8* NIVs in sporadic HA; (b) DNVs in NIV sporadic HA occur not only in gonial cells but also in the first few cell divisions of the zygote stage [12,13], as well as in early postzygotic development [12,13]; the variant rate may correlate with the number of cell divisions; (c) the family member designated as the DNV origin must be free of coagulation alteration and genetic variants related to DNV; (d) DNV origin and occurrence are distinct events.

In this study, we conducted a direct clinical determination of sex differences in the origin of sporadic HA-NIV according to data from two families with HA-NIV in addition to the data presented in our recent study [12], but we did not include the sex difference in the origin of NIV among the reported results. Several previous studies in the literature attempted to estimate the sex ratio of the mutation frequency for sporadic HA [9,14,15,16,17,18]. Most of these previous studies reported male predominance [9,16,17,18], whereas a few studies [14,15] revealed an equal sex ratio. Notably, these studies had several caveats; it was unclear whether they studied the origin of sporadic HA or the occurrence of sporadic HA variants. Additionally, the studies combined inversion and noninversion variants, even after 1993, when inversion variants were determined [19,20,21]. The sex ratio of mutation frequency of each variant type may be quite different, and previous studies were based on the assumption that variants occurred mainly in the gonadal cell stage but not in the postzygotic stage, yet this was not consistent with several other reports [12,13,22,23,24]. Finally, sporadic HA (DNV-HA) was not well defined. We revealed that our results differed compared to previous studies based on our direct clinical study data.

This study aims to (a) demonstrate by direct clinical data study that HA sporadic NIV may originate mostly from female family members and (b) improve knowledge of the genetic pathogenesis of HA. We confirmed that HA sporadic NIVs originate mostly in females. This result contributes a better understanding of the genetic pathogenesis in HA.

## 2. Results

### 2.1. Clinical and Genetic Characteristics of Patients with Sporadic Noninversion HA

This study only included 24 of 126 registered families because the inclusion criteria required families to have both sporadic HA and NIV. Additionally, obtaining samples from some family members was difficult. Data from families 1–22 have been reported in recent studies [12] (Tables S1 and S2). Two newly determined families included families 23 and 24. The proband of family 23 was a patient with severe HA with variant c.3637delA, p.I1213Ffs*5, aged 1 year, whereas the proband of family 24 was a patient with mild HA (FVIII: C 7.8 IU/dL) with variant c.6506G>A, p.R2169H, aged 54 years. Table 1 summarizes that nineteen, three, and two patients were diagnosed with severe, moderate, and mild HA, respectively. The ages of the patients ranged from 6 days to 57 years, with a mean and median of 16.8 years and 10 years, respectively. The genetic variants determined included thirteen point variants (of which nine were missense variants and four were nonsense variants), two splice-site variants, four duplications, and five deletions.

### 2.2. Identifying the Origin of Sporadic HA by Linkage Analysis, Genetic Testing, and Amplification Refractory Mutation System–Quantitative Polymerase Chain Reaction (ARMS-qPCR)

#### 2.2.1. Analysis and Testing in Families 1 to 19, 23, 24, Who Have Sporadic HA-NIVs Without Mosaic Variants

**Figure 1:**
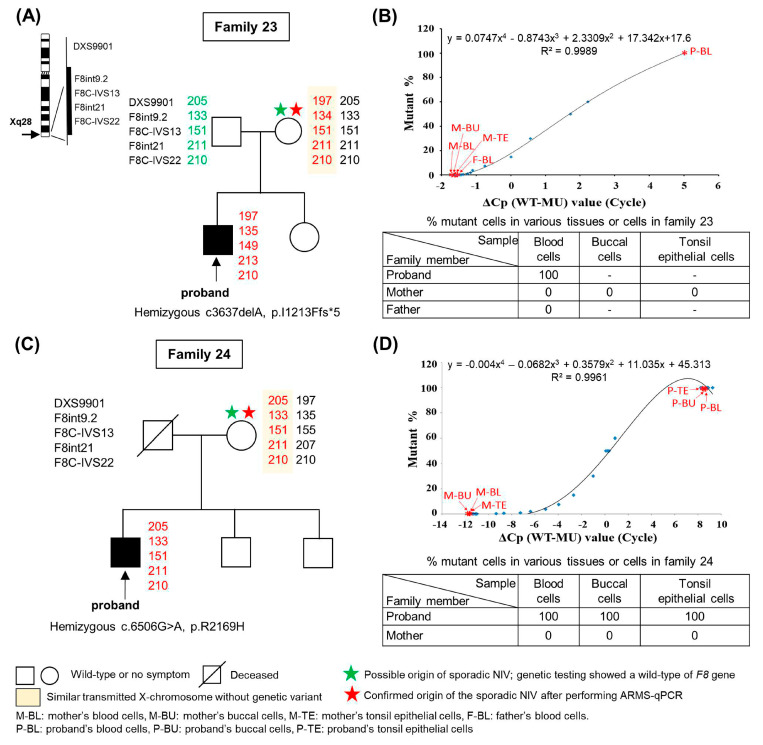
Linkage analysis using intra- and extragenic markers for the *F8* gene, genetic analysis, and amplification refractory mutation system–quantitative polymerase chain reaction (ARMS-qPCR) to confirm the origin of the sporadic noninversion variant (NIV) in families 23 (severe hemophilia A) and 24 (mild hemophilia A), respectively (**A**,**C**). The *F8* gene located on the X-chromosome (Xq28) is illustrated on the left side of (**A**), along with the four intragenic markers (F8int9.2, F8IVS13, F8int21, and F8IVS22) and one extragenic marker (DXS9901) selected for linkage analysis. (**B**,**D**) Determination of the percentage of mutant cells with the ARMS-qPCR in various tissue cells collected from family members to confirm the origin of the sporadic NIVs (~0% mutant cells) in families 23 and 24, respectively. ΔCp (wild-type [WT] − mutant [MU]) indicates the differences between the qPCR cycle crossing points (Cp) of the WT and MU alleles of different synthetic dilutions (X-axis). Blue points (◆) indicate various synthetic dilutions prepared via a twofold serial dilution of MU DNA using WT DNA, as described in the text. The standard curve presented in the upper part of each figure was derived by plotting the ΔCp value (X-axis) against known mutant percentages of various synthetic dilutions (Y-axis). Thereafter, the equations for X and Y were generated. Y (% mutant cells) could be calculated from each equation, as shown in each table, when the ΔCp (X) of the test sample after ARMS-qPCR was known (shown by red asterisks).

Linkage analysis and ARMS-qPCR were conducted in tissue cells obtained from various family members in each family. Table S1 summarizes the results of previous families studied (families 1–19). Therefore, only results from the two new families (families 23–24) will be presented. Figure 1A illustrate that the mother of family 23, whose five *F8* markers were of a similar size to that of the proband (shown in red), was considered to have the same transmitted X chromosomes. Further genetic testing revealed a wild-type (WT) *F8* allele derived from the mother, which would be, therefore, designated as the possible origin of the sporadic NIV (indicated by a green star). Further ARMS-qPCR was performed in family members of the proband, and results shown in the small table in Figure 1B reveal that the percentage of mutant (MU) cells was approximately 100% in the blood cells of the proband, as expected, whereas 0% MU cells in various tissue cells were found in the mother. These results confirm that the sporadic NIV of the proband originated from the mother (indicated by a red star). Similar test results as described above were found in family 24, as illustrated in Figure 1C,D, that the mother was designated as the possible origin of the sporadic NIV by linkage analysis and genetic testing (Figure 1C), followed by confirmation as the origin of the sporadic NIV by ARMS-qPCR (Figure 1D).

#### 2.2.2. Analysis and Testing in Families 20–22, Who Had Mosaic Variants

The mother in three determined families may be the origin of a sporadic NIV based on both linkage analysis and Sanger genetic testing. However, ARMS-qPCR revealed some MU cells in various tissue cells of three mothers, indicating that three mothers demonstrated mosaic variants. Further linkage analysis, genetic testing, and ARMS-qPCR confirmed that the maternal grandmother (MGM) of family 20, maternal grandfather (MGF) of family 21, and earlier generation than mother (EGT, M) of family 22 were the origins of the sporadic NIV, respectively. A previous study described these results (Table S2) [12].

#### 2.2.3. Analysis and Testing in Carrier Mothers of Families 10–19, in Which the Confirmed Origins of Sporadic NIVs Were Designated as Maternal Grandparents (MGPs)

MGPs of families 10–19 were designated as the confirmed origins of sporadic NIVs, as shown in Table S1; their descendants (mother) become HA carriers. The percentage of mutant cells obtained by ARMS-qPCR in blood cells and buccal cells all revealed approximately 50%, as expected. Only two mothers (Table 2, families 12 and 15) had their tonsil epithelial cells tested, showing mutant cells of 50.6% and 50.3%, respectively. These data suggest that all carrier mothers had *F8* gene heterozygous variants.

### 2.3. Sex Differences in the Origin of Noninversion Variants in 23 Families with Sporadic HA

Table 1 presents that eleven mothers (Table 1, families 1–9, 23, 24), five MGMs (Table 1, families 10–13, 20), and seven MGFs (Table 1, families 14–19, 21) were determined as the origin of the sporadic NIV based on our criteria for the origin of sporadic NIVs. The origin of the sporadic HA-NIV in family 22 (EGT M) could not be determined. The female-to-male ratio of the origin in sporadic events 1 (the MGP group) and 2 (the mother group) would be 5:7 and 11:0, respectively. Fisher’s exact test revealed a highly significant difference in the female-to-male distribution between the two sporadic event groups (*p* = 0.005). The proportion of females vs. males in the total group was sixteen (16/23, 69.6%) vs. seven (7/23, 30.4%), respectively.

## 3. Discussion

Sporadic patients were recruited among all the registered families with HA in our hemophilia center to avoid selection bias. However, only 23 families were available for the study, as the inclusion criteria required sporadic HA families with NIVs, and there were also difficulties in obtaining samples from eligible family members. We defined that the origin of sporadic NIVs differs from sporadic NIV occurrences, regardless of variant, whether a complete or undetectable mosaic, as mentioned in the definition criteria of the origin, so that the origin of sporadic NIVs can be correctly studied. ARMS-qPCR, which demonstrates a better variant detection sensitivity (<0.1%) [25,26], was performed on three germ layer-derived tissue cells and confirmed the origin of sporadic HA-NIVs to avoid misdiagnosing their origins [12].

Most of the previous reports of sex ratio in the origin of sporadic HA used a formula calculation method, so not only was the origin of the sporadic variant not distinctly defined, but the study was also based on the concept that the sporadic variant occurred in gametes and depended on assumption of the genetic equilibrium, a balance between mutation and selection, and an estimate of the patient’s fitness. As we previously mentioned, variants occur in the germ cells as well as in the postzygotic stage, especially during the first few cell divisions and the early zygotic phase [12,13], although previous studies have neglected the latter. Spermatogonial stem cells divide via mitosis approximately every 16 days throughout the lifespan of male individuals, both maintaining their pool and producing sperm cells via meiosis, whereas oogonia stop dividing soon after birth and only meiosis, which occurs during ovulation, is observed throughout the reproductive lifespan of female individuals [23,27]. A higher ratio in male individuals would be observed if the sex ratio estimation depended on the assumption that sporadic variants occurred in egg and sperm gametes. Estimating genetic equilibrium is difficult, and patient fitness is changing during the present era of advanced hemophilia care [9]. The variant rate is generally correlated with the number of cell divisions. The highest number of cell divisions occurs during spermatogenesis throughout male reproductive life, and the lowest number of cell divisions occurs during oogenesis [27,28]. Over 90% of *F8* inversions, which are present in 40–50% of HA sporadic *F8* variants [11,20], occur during spermatogenesis. Admixing of *F8* inversion and noninversion variants together to estimate sex differences in the origin of variants may explain why the sex ratios of mutation frequency in sporadic HA in previous studies all exhibited higher rates from male individuals [9,16,17,18,19,20] and only a few demonstrated equal frequency [14,15]. Some previous formula calculation studies [9,19] revealed the direct estimation of the sex ratio of mutation frequency by comparing the probability of a new mutation in one of the MGF’s cells, with the probability of a de novo mutation occurring in a cell of the patient’s MGM (MGF/MGM) and mother (M) (MGF/M), respectively, but not MGM and mother together, i.e., MGF/MGM+M; the sex ratio obtained was not the real male/female ratio. Furthermore, the MGF was no longer relevant to the origin of the sporadic NIV if the mother was proven to be the origin of the NIV-DNV, and thus the MGF/M calculation was meaningless.

We determined two sporadic HA-DNV events. One event revealed that the origins of DNVs were from MGPs, and the occurrence of DNVs was in mothers (carrier mothers), who then gave birth to the probands. The other event revealed that the origin of DNVs was from mothers exclusively and the occurrence of DNVs took place in the probands. A female-to-male ratio of 2:1 in the genetic variation rate would be expected in a three-generation family; if the mother was a sporadic HA carrier, then the MGM provided two X chromosomes compared to one single X chromosome derived from the MGF. However, the higher number of cell divisions in spermatogenesis may compensate for the female-to-male ratio in the genetic variation rate. Our results revealed that five MGMs and seven MGFs were the origins of sporadic HA; thus, these data indicate that spermatogenesis has a 2.8 (2:1 ratio becomes 5:7 ratio) times higher genetic variation rate, consistent with the previous reports that observed a male-to-female mutation ratio of 2–7 [27,29,30]. The mother will be the only contributor of an X chromosome if the mother is determined to be the exclusive origin of the HA-sporadic NIV. In general, more mothers are willing to participate in the examinations of studies than MGPs. The number of mothers who join the study and are determined to be the origin of the sporadic HA will affect the sex difference in the origin of the sporadic HA-NIV. Nevertheless, in contrast to the previous studies, our direct clinical data study reveals that sporadic HA-NIVs originate mostly from females (69.6%) rather than from males (30.4%). Furthermore, we consider that the timing of the occurrence of sporadic HA-NIVs in the female (MGM and mother) was most likely within the first few cell divisions during the embryogenesis of their respective offspring, i.e, carrier mother and proband, respectively, but not during their oogenesis, whereas the timing of the occurrence of the sporadic HA-NIV in the MGF was most likely during spermatogenesis. These considerations have been clearly presented in our recent report [12].

We are confident that the concepts and methodology we have used were right. However, some limitations indicate that almost no germinal tissues are available, especially from female family members, to show germline genetic variants or isolated germline mosaicism. In addition, a relatively small number of families were studied. Thus, more sporadic hemophilia families are required for future studies. Two sporadic DNV events were determined in this study; one event occurred in grandparents and the other event was identified in mothers. If more than one carrier mother in the first event or more than one patient in the second event were identified, respectively, these findings would highly suggest that germline genetic variants or isolated germline mosaicism occur in the grandparents or mother, respectively.

## 4. Materials and Methods

### 4.1. Patients and Family Groups and Study Design

This study was conducted under the Declaration of Helsinki and approved by the Institutional Review Board of Changhua Christian Hospital (approval No. 201209). Only patients who bore HA sporadic NIVs were investigated. All patients and relevant family members provided informed consent. Sporadic patients were recruited from 2015 to 2024 from among 126 registered families with HA to prevent selection bias. Most families contain three generations of data (patients to MGPs). Several patients were excluded for various reasons, consisting of familial (*n* = 28), unknown history (*n* = 15), unknown genetic variants (*n* = 8), and inversion variants (*n* = 27). A few families were excluded because of their unwillingness to participate in the study (*n* = 2), insufficient data (*n* = 1), or the unavailability of patients’ mothers or MGPs for blood sampling (*n* = 22). The last situation had two exceptions: (a) only one of the MGPs was available for blood sampling and he/she was confirmed as the origin of the genetic variants and (b) the mother was confirmed as the origin of the genetic variant, so further study in the MGPs was not required.

Thus, 23 patients—excluding family 22, the origin of which was considered to be EGT M—and their family members were eligible for complete studies, including linkage analysis and genetic testing, to determine the possible origin of sporadic NIVs. A custom-designed technique, namely ARMS-qPCR, was performed to confirm the real origin of the sporadic NIVs (~0% MU cells in various tissue cells). The parental generation were further tested to determine the true origin of the sporadic NIV, if the relevant family members were identified as possessing an *F8* mosaic variant by ARMS-qPCR, with 0 to 50% MU cells in various tissue cells. Finally, sex differences between the origins of sporadic HA variants were determined.

### 4.2. DNA Extraction from Blood and Tissue Cells

DNA extraction from the blood and tissue cells of patients and family members was performed as described elsewhere [12,22]. Blood, buccal, and palatine tonsil epithelial cells are derivatives of the mesoderm, ectoderm, and endoderm, respectively [31,32]. The DNA isolated from these tissue cells, or sperm cells if available, were utilized for F8 genetic analysis and percentage of MU cell detection by ARMS-qPCR, as described below (the method is described at subtitle 4.5 ARMS-qPCR, using the proband of family 23 as an example; all designed primers used for all families 1–24 are shown in Table S3).

### 4.3. Laboratory Diagnosis of HA

Coagulation testing and genetic analysis for HA diagnosis were performed as previously described [12,33,34]. The nomenclature for the description of sequence variants follows the guidelines of the Human Genome Variation Society (https://hgvs-nomenclature.org/, accessed on 10 August 2022).

### 4.4. Identification of the Possible Origin of Sporadic NIVs by Linkage Analysis and Genetic Testing

*F8* is X chromosome-linked; thus, linkage analysis using intra- and extragenic HA gene markers could determine the possible origin of sporadic NIVs in the parental or grandparental generation. Some selected intragenic markers of HA are *F8*int9.2, *F8*IVIS13, *F8*int21, and *F8*IVIS22, and DXS9901 is the extragenic maker. Linkage analysis was performed as previously reported [12,35,36]. A family member who was determined as the origin of a sporadic NIV, as identified by linkage analysis, and demonstrated a WT *F8*, as detected by genetic testing, would be considered as the possible origin of the sporadic NIV.

### 4.5. ARMS-qPCR

**Figure 2:**
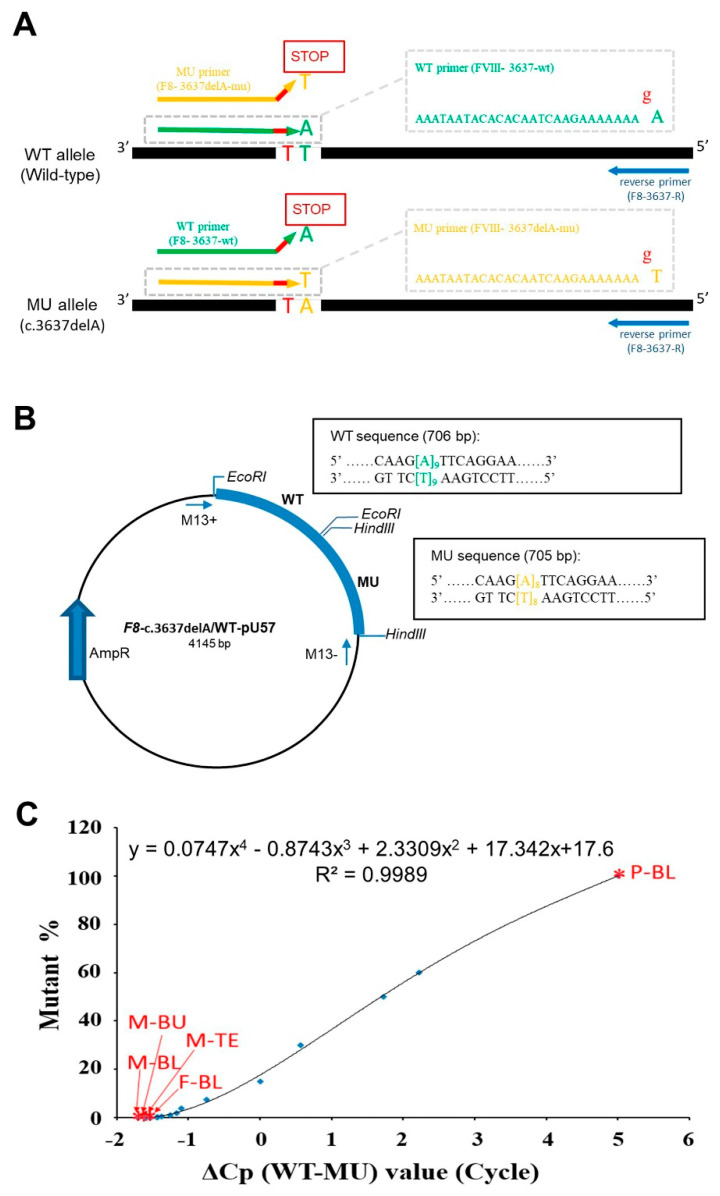
Schematic overview of the ARMS-qPCR process for detecting the FVIII c.3637delA variant in family 23. (**A**) ARMS-qPCR setup for determining both mutant (MU) and wild-type (WT) alleles using allele-specific primers with distinct forward primers for mutant (MU) and wild-type (WT) alleles. A mismatch (red “g”) was introduced at the second-to-last nucleotide of the variant to enhance amplification specificity. (**B**) Synthetic plasmid (*F8*-c.3637delA/WT-pU57) construction on a pUC57 backbone with AmpR, containing MU and WT sequences inserted via EcoRV. Accuracy was validated by bidirectional sequencing (M13+ and M13− primers) and restriction sites (EcoRI and HindIII) flanking the sequences. (**C**) Standard curve used for ARMS-qPCR analysis. The equation relates the ΔCp (difference in crossing points between WT and MU) (X) to the percentage of mutant cells (Y), which were generated from 2-fold serial dilutions and calibrated with the synthetic plasmid. Mutant cell proportions were calculated from the ΔCp values of test samples (red asterisks). Abbreviations: P-BL (proband blood), F-BL (father blood), M-BL (mother blood), M-BU (mother buccal cells), M-TE (mother tonsil epithelial cells).

The ARMS-qPCR technique is a specialized form of PCR that enables differential amplification of MU and WT alleles, providing heightened sensitivity for detecting low MU cell levels. Given its precision, we used the ARMS-qPCR method to investigate the familial origin of genetic variants. To achieve this, two sequence-specific forward primers were established with a mismatch at the penultimate nucleotide position of the variant site. In particular, the analysis of family 23, which carried the c.3637delA mutation in the *F8* gene (as illustrated in Figure 2A), used the following primers: F8-3637delA-mu (5′-AAATAATACACACAATCAAGAAAAAAAGT-3′) for the MU allele and F8-3637-wt (5′-AAATAATACACACAATCAAGAAAAAAAGA-3′) for the WT allele. Both forward primers were paired with the reverse primer F8-3637-R (5′-TGAGGCAAAACTACATTCTCTTGG-3′), producing 86 bp and 87 bp products, respectively. Table S3 comprehensively lists the primers used for each family.

The DNA of the male proband (100% MU) and a healthy male (100% WT) were selected, and serial dilutions were performed to generate the samples required for the standard curve after the confirmation by family sequencing analysis. Additionally, a synthetic PUC57 plasmid (Protech Technology Enterprise Co., Taipei, Taiwan) containing WT and MU *F8* sequences (representing 50% variation) was utilized as a calibration standard (Figure 2B), which was appropriately diluted to optimize the subsequent curve generated by qPCR.

All DNA samples, consisting of serially diluted standards, family member samples, and the synthetic plasmids, were subjected to the same ARMS-qPCR experiment. The experiments were conducted in triplicate with a Light Cycler^®^ 480 Real-Time PCR System (Roche, Rotkreuz, Switzerland) and performed in 20-µL reactions with 0.5 ng of each DNA sample, 0.5 μmol/L of each primer, and 1× Smart Quant Green Master Mix (Protech Technology Enterprise Co., Taipei, Taiwan). Cycling conditions were 10 min at 95 °C to denature the DNA, followed by cycles of amplification at 95 °C for 15 s and 60 °C for 60 s, with primers selectively binding MU or WT sequences according to mismatch sensitivity. Amplification of either the WT or MU allele was monitored during qPCR. Heterozygous samples will exhibit amplification of both alleles, whereas homozygous MU or WT samples will demonstrate amplification of only one set of primers. The proportion of MU versus WT alleles was calculated from the amplification data with the LightCycler 480 software. The results were analyzed by comparing the standard curve (Figure 2C).

## 5. Conclusions

We conducted a direct clinical data study to identify the sex difference in the origin of sporadic HA-NIVs. We determined two sporadic events and revealed that the origin originated from a majority of female family members at 69.6% compared to 30.4% from male family members. The timing of occurrence of the sporadic HA-NIV mutation in females (MGM and mother) was considered most likely within the first few cell divisions during the embryogenesis of their respective offspring (carrier mother and proband, respectively), whereas the timing of the occurrence of the sporadic HA-NIV mutation in males (MGP) was considered most likely during their spermatogenesis. These studies help better understand the genetic pathogenesis of HA and improve genetic counseling for this disease.

## Figures and Tables

**Table 1:** Sex differences in the origin of the noninversion variant (NIV) of the *F8* gene in sporadic hemophilia A.

Family No.	FVIII Level (IU/dL)	Nucleotide Change	Amino Acid Substitution	Family Members Designated as the Origin of Sporadic NIV ^§^
1	<1	c.6046C>G	p.R2016G	M
2	<1	c.822G>T	p.W274C	M
3	<1	c.1648C>T	p.R550C	M
4	3.7	c.5122C>T	p.R1708C	M
5	<1	c.6131T>C	p.L2044P	M
6	1	c.4379delA	p.N1460Ifs*5	M
7	<1	c.1412T>A	p.L471*	M
8	<1	c.403G>A	p.D135N	M
9	<1	c.2945dupA	p.N982Kfs*9	M
10	<1	c.2945dupA	p.N982Kfs*9	MGM
11	<1	c.5343T>A	p.Y1781*	MGM
12	<1	c.3637delA	p.I1213Ffs*5	MGM
13	1.2	c.1538-1G>A	-	MGM
14	<1	c.1848dupT	p.P617Sfs*7	MGF
15	<1	c.5219+1G>A	-	MGF
16	<1	c.1813T>C	p.Y605H	MGF ^†^
17	<1	c.2322delA	p.Q774Hfs*12	MGF
18	<1	c.3637dupA	p.I1213Nfs*28	MGF
19	<1	c.6548_6554delTGGAGTT	p.M2183Rfs*9	MGF
20	<1	c.1525A>T	p.R509*	MGM ^‡^
21	25.1	c.1636C>T	p.R546W	MGF ^‡^
22	<1	c.185 C>G	p.S62*	EGT M ^‡^
23	<1	c.3637delA	p.I1213Ffs*5	M
24	7.8	c.6506G>A	p.R2169H	M

M: mother; MGM: maternal grandmother; MGF: maternal grandfather; EGT, earlier generation than; ^§^ tissue cells (blood cells, buccal cells, and tonsil epithelial cells) obtained from each of them all demonstrated 0% mutant cells by amplification refractory mutation system–qualitative polymerase chain reaction; ^†^ sperm cells also exhibited 0% mutant cells; ^‡^ their descendants (mother) demonstrated mosaic variants. The data of family numbers 1–22 are shown in Supplementary Tables S1 and S2.

**Table 2:** Characteristics of carrier mothers of families with the hemophilia A sporadic noninversion variant (NIV) and their percentage of mutant cells, as determined by amplification refractory mutation system–quantitative polymerase chain reaction (ARMS-qPCR) in the tissue cells obtained.

Family No. (Age)	Nucleotide Change	Amino Acid Substitution	Family Members Designated as the Confirmed Origin of Sporadic NIVs	Percentage of Mutant Cells Obtained by ARMS-qPCR in Tissue Cells from Carrier Mothers of Families with Hemophilia A Sporadic NIVs		
				Blood Cells	Buccal Cells	Tonsil Epithelial Cells
10 (55)	c.2945dupA	p.N982Kfs*9	MGM	42.6	46.6	NA
11 (42)	c.5343T>A	p.Y1781*	MGM	47.7	47.5	NA
12 (49)	c.3637delA	p.I1213Ffs*5	MGM	50.4	49.3	50.6
13 (33)	c.1538-1G>A	-	MGM	47.2	45.6	NA
14 (27)	c.1848dupT	p.P617Sfs*7	MGF	45.8	46.5	NA
15 (32)	c.5219+1G>A	-	MGF	47.7	47.2	50.3
16 (25)	c.1813T>C	p.Y605H	MGF ^†^	46.5	51.4	NA
17 (31)	c.2322delA	p.Q774Hfs*12	MGF	42.6	51.4	NA
18 (35)	c.3637dupA	p.I1213Nfs*28	MGF	50.3	51	NA
19 (37)	c.6548_6554delTGGAGTT	p.M2183Rfs*9	MGF	43.9	45.5	NA

MGM, maternal grandmother; MGF, maternal grandfather; NA, not available; ^†^ sperm cells also exhibited 0% mutant cells.

## Data Availability

The data reported in this study are available upon request from the corresponding author. These data are not publicly available due to ethical restrictions.

## Supplementary Materials

The following supporting information can be downloaded at https://www.mdpi.com/article/10.3390/ijms26030891/s1.
