# Supplementary material for: Noninversion Variants in Sporadic Hemophilia A Originate Mostly from Females
Source: Int J Mol Sci. 2025 Jan 22;26(3):891. doi: 10.3390/ijms26030891 (PMC11816929; doi:10.3390/ijms26030891)
Supplement: Supplementary file 1 [file ijms-26-00891-s001.zip › ijms-3397049-supplementary.pdf]

Supplemental Table S1. Characteristics of the proband and percentage of mutant cells as determined by amplification refractory mutation system–quantitative polymerase chain reaction (ARMS-qPCR) in tissue cells obtained from family members designated as a possible and confirmed origin of hemophilia A sporadic noninversion variant (NIV).

Family No. and Age of Proband (Year)	Family File No.	FVIII Level (IU/dL)	Exon	Nucleotide Change	Amino Acid Substitution	Family Members Designated as the Possible Origin of Sporadic NIVs	Percentage of Mutant Cells by ARMS-qPCR in Tissue Cells Obtained from Family Members Designated as the Possible Origin of Sporadic NIVs			Family Members Designated as the Confirmed Origin of Sporadic NIVs	Percentage of Mutant Cells by ARMS-qPCR in Tissue Cells Obtained from Family Members Designated as Confirmed Origin of Sporadic NIVs		
							Blood Cells	Buccal Cells	Tonsil Epithelial Cells		Blood Cells	Buccal Cells	Tonsil Epithelial Cells
1 (16)	74	<1	19	c.6046C>G	p.R2016G	M	0	0	0	M	0	0	0
2 (57)	31	<1	7	c.822G>T	p.W274C	M	0	0	0	M	0	0	0
3 (9)	189	<1	11	c.1648C>T	p.R550C	M	0	0	0	M	0	0	0
4 (15)	176	3.7	14	c.5122C>T	p.R1708C	M	0	0	0	M	0	0	0
5 (6)	208	<1	20	c.6131T>C	p.L2044P	M	0	0	0	M	0	0	0
6 (11)	209	1.0	14	c.4379delA	p.N1460Ifs*5	M	0	0	0	M	0	0	0
7 (48)	NTUH	<1	9	c.1412T>A	p.L471*	M	0	0	0	M	0	0	0
8 (26)	147	<1	4	c.403G>A	p.D135N	M	0	0	0	M	0	0	0
9 (4)	180	<1	14	c.2945dupA	p.N982Kfs*9	M	0	0	0	M	0	0	0
10 (23)	referred-1	<1	14	c.2945dupA	p.N982Kfs*9	MGM	0	0	0	MGM	0	0	0
11 (22)	155	<1	15	c.5343T>A	p.Y1781*	MGM	0	0	0	MGM	0	0	0
12 (19)	78	<1	14	c.3637delA	p.I1213Ffs*5	MGM	0	0	0	MGM	0	0	0
13 (7)	131	1.2	IVS10	c.1538-1G>A	-	MGM	0	0	0	MGM	0	0	0
14 (2)	207	<1	12	c.1848dupT	p.P617Sfs*7	MGF	0	0	0	MGF	0	0	0
15 (0.1)	0	<1	IVS14	c.5219+1G>A	-	MGF	0	0	0	MGF	0	0	0
16 (5)	referred-2	<1	12	c.1813T>C	p.Y605H	MGF+	0	0	0	MGF+	0	0	0
17 (5.4)	187	<1	14	c.2322delA	p.Q774Hfs*12	MGF	0	0	0	MGF	0	0	0
18 (2)	NTUH-2	<1	14	c.3637dupA	p.I1213Nfs*28	MGF	0	0	0	MGF	0	0	0
19 (7)	211	<1	23	c.6548_6554delTGGAGTT	p.M2183Rfs*9	MGF	0	0	0	MGF	0	0	0

M, mother ; MGM, maternal grandmother; MGF, maternal grandfather. + Sperm exhibited 0% mutant cells

Supplemental Table S2. Characteristics of the proband and percentage of mutant cells as determined by amplification refractory mutation system–quantitative polymerase chain reaction (ARMS-qPCR) in tissue cells obtained from family members designated as a possible and confirmed origin of hemophilia A sporadic mosaic noninversion variant (NIV).

Family No. and Age of Proband (Year)	Family File No.	FVIII Level (IU/dL)	Exon	Nucleotide Change	Amino acid Substitution	Family Members Designated as the Possible Origin of Sporadic NIVs	Percentage of Mutant Cells by ARMS-qPCR among Tissue Cells Obtained from Family Members Designated as the Possible Origin of Sporadic Mosaic NIVs			Family Members Designated as the Confirmed Origin of Sporadic NIVs	Percentage of Mutant Cells by ARMS-qPCR among Tissue Cells Obtained from Family Members Designated as Confirmed Origin of Sporadic Mosaic NIVs		
							Blood Cells	Buccal Cells	Tonsil Epithelial Cells		Blood Cells	Buccal Cells	Tonsil Epithelial Cells
20 (10)	153	<1	10	c.1525A>T	p.R509*	M	18.1	23.8	24.1	MGM	0	0	0
21 (38)	130	25.1	11	c.1636C>T	p.R546W	M	7.3	5.1	2.8	MGF	0	0	0
22 (47)	105	<1	2	c.185 C>G	p.S62*	M	9.4	3.7	5.4	EGT M	NA	NA	NA

M, mother; MGM, maternal grandmother; MGF, maternal grandfather; EGT, earlier generation than; NA: not available.

Supplemental Table S3. Primer pairs used for ARMS-qPCR

Family No.	Nucleotide change	Forward primer	Sequence 5′→ 3′	Tm	Reverse primer	Sequence 5′→ 3′	Tm	size (bp)	Genomic position (hg38)
1	F8 (c.6046C>G)	F8-6046C-wt	CCATCCAAAGCTGGAATTTG**A**C	55.1	F8-6046-R	GAACCTCTGCCCACATTGCTAC	55.2	117	>chrX:154902025-154902141
		F8-6046G-mu	CCATCCAAAGCTGGAATTTG**A**G	55.3					
2	F8 (c.822G>T)	F8-822G-wt	GCCACAGGAAATCAGTCTAT**C**GG	56.5	F8-822-R	CCAAGGTCCATCAAGAGTGTTTG	55.1	169	>chrX:154969372-154969540
		F8-822T-mu	GCCACAGGAAATCAGTCTAT**C**GT	54					
3	F8 (c.1648C>T)	F8-1648C-wt	CAGATCCTCGGTGCCTGAC**T**C	56	F8-1648-R	CAGCACTTGGAAAGGCAAGAAC	55.5	151	>chrX:154956931-154957081
		F8-1648T-mu	CAGATCCTCGGTGCCTGAC**T**T	55.6					
4	F8 (c.5122C>T)	F8-5122-F	GGCAAAGCAAGGTAGGACTGAA	55.1	F8-5122C-wt	GTCGTGTTTTCTTTTGAAAGCTG**T**G	56.2	212	>chrX:154928644-154928855
					F8-5122T-mu	GTCGTGTTTTCTTTTGAAAGCTG**T**A	54		
5	F8 (c.6131T>C)	F8-6131T-wt	ATTTCAGAGTGTCAGACTCCC**G**T	53.5	F8-6131-R	ATAATCAGCCCAGGTTCTTGGAG	55.4	121	>chrX:154901329-154901449
		F8-6131C-mu	ATTTCAGAGTGTCAGACTCCC**G**C	56.2					
6	F8 (c.4379delA)	F8-4379-wt	TTTCTTACAAGGAGCCAAAAAA**G**AT	54.1	F8-4379-R	AGATGTTTTGGGCAAGTCTGGTT	55.7	168	>chrX:154929266-154929434
		F8-4379delA-mu	TTTCTTACAAGGAGCCAAAAAA**G**TA	53.1				167	

7	F8 (c.1412T>A)	F8-1412T-wt	GAATCAGGAATCTTGGGACCT**C**T	54.3	F8-1412-R	CCATTGGAGACAAGGCTGAATTA	54.9	151	>chrX:154965873-154966023
		F8-1412A-mu	GAATCAGGAATCTTGGGACCT**C**A	55.3					
8	F8 (c.403G>A)	F8-403G-wt	CCTGCTATAGGAGCTGAATATGA**A**G	52.9	F8-403-R	TAGGTAAGGCACAGTGGGTCAG	53.4	146	>chrX:154993013-154993158
		F8-403A-mu	CCTGCTATAGGAGCTGAATATGA**A**A	53.2					
9	F8 (c.2945dupA)	F8-2945A-wt	CAAGAAAGTTCATGGGGAAAAA**G**T	54.7	F8-2945-R	AGTCAACAAAGCAGGTCCATGAG	54.9	90	>chrX:154930778-154930867
		F8-2945dupA-mu	CAAGAAAGTTCATGGGGAAAAA**G**A	55.5				91	
10	F8 (c.2945dupA)	F8-2945A-wt	CAAGAAAGTTCATGGGGAAAAA**G**T	54.7	F8-2945-R	AGTCAACAAAGCAGGTCCATGAG	54.9	90	>chrX:154930778-154930867
		F8-2945dupA-mu	CAAGAAAGTTCATGGGGAAAAA**G**A	55.5				91	
11	F8 (c.5343 T>A)	F8-5343T-wt	GGACTCCTGGGGCCAT**G**T	54.1	F8-5343-R	CCACTGTCCTTAACTCACCATGATA	54	66	>chrX:154906402-154906467
		F8-5343A-mu	GGACTCCTGGGGCCAT**G**A	55.2					
12	F8 (c.3637delA)	F8-3637-wt	AAATAATACACACAATCAAGAAAAAAA**G**A	53	F8-3367-R	TGAGGCAAAACTACATTCTCTTGG	54.4	87	>chrX:154930095-154930181
		F8-3637delA-mu	AAATAATACACACAATCAAGAAAAAAA**G**T	52.3				86	
13	F8 (c.1538-1 G>A)	F8-1538-1G-wt	TATGGTTTTGCTTGTGGGT**G**G	54.8	F8-1538-R	TGAGGAGAGGGCCAATGAGT	53.4	198	>chrX:154956995-154957192
		F8-1538-1A-mu	TATGGTTTTGCTTGTGGGT**G**A	53					

14	F8 (c.1848dupT)	F8-1848-wt	TACAACGCTTTCTCCCCAA**C**C	55.8	F8-1848-R	ATGTTGGAGGCTTGGAACTCTG	54.8	67	>chrX:154953900-154953966
		F8-1848dupT-mu	TACAACGCTTTCTCCCCAA**C**T	53.1					
15	F8 (c.5219+1G>A)	F8-5219+1F	AGTGGAGAGGCTCTGGGATTATG	55.4	F8-5219+1G-wt	AAAGGAATAACCAATGCATTCAT**G**C	57.3	82	>chrX:154928546-154928627
					F8-5219+1A-mu	AAAGGAATAACCAATGCATTCAT**G**T	54.9		
16	F8 (c.1813T>C)	F8-1813T-wt	ATTTGATGAGAACCGAAGCTG**A**T	53.7	F8-1813-R	TTCTTTATTCACCACCCACTGGA	55	157	>chrX:154953848-154954004
		F8-1813C-mu	ATTTGATGAGAACCGAAGCTG**A**C	54.1					
17	F8 (c.2322delA)	F8-2322-wt	CCTAGCACTAGGCAAAAGC**G**A	54.1	F8-2322-R	TGTCGCAAGAGCATCAACAAAT	54.8	143	>chrX:154931346-154931488
		F8-2322delA-mu	CCTAGCACTAGGCAAAAGC**G**T	53.3				142	
18	F8 (c.3637dupA)	F8-3637-wt	AAATAATACACACAATCAAGAAAAAAAA**G**T	53.5	F8-3367-R	GCCCCGTCATATGAACCTTCTAC	55.6	177	>chrX:154930005-154930181
		F8-3637dupA-mu	AAATAATACACACAATCAAGAAAAAAAA**G**A	54.1					
19	F8 (c.6548_6554delTGGAGTT)	F8-6548-wt	CGCAGCACTCTTCGC**G**TG	56.3	F8-6548del7-R	CTACCCATGGTTGAGGGAAGAAG	55.7	90	>chrX:154863036-154863125
		F8-6548del7-mu	CGCAGCACTCTTCGC**G**GA	58.2					

20	F8 (c.1525 A>T)	F8-1525A-wt	GATGTCCGTCCTTTGTATTCAAG**A**A	55.4	F8-1525-R	ACAGCTGGAGAAAGGACCAACA	55.2	139	>chrX:154960973-154961111
		F8-1525T-mu	GATGTCCGTCCTTTGTATTCAAG**A**T	54.5					
21	F8 (c.1636C>T)	F8-1636C-wt	GGGCCAACTAAATCAGATCC**A**C	54.1	F8-1636-R	GATGAGGAGAGGGCCAATGAGT	55.9	102	>chrX:154956993-154957094
		F8-1636T-mu	GGGCCAACTAAATCAGATCC**A**T	53.7					
22	F8 (c.185C>G)	F8-185C-wt	AAATCTTTTCCATTCAACACC**C**C	55.1	F8-185-R	CCTTGGCTTAGCGATGTTGAAA	56	90	>chrX:154999492-154999581
		F8-185G-mu	AAATCTTTTCCATTCAACACC**C**G	56					
23	F8 (c.3637delA)	F8-3637-wt	AAATAATACACACAATCAAGAAAAAAA**G**A	53	F8-3637-R	TGAGGCAAAACTACATTCTCTTGG	54.4	87	>chrX:154930095-154930181
		F8-3637delA-mu	AAATAATACACACAATCAAGAAAAAAA**G**T	52.3					
24	F8 (c.6506G>A)	F8-6506G-wt	CCAATTATTGCTCGATACATC**G**G	55.3	F8-6506-R	AGGATATGGGATGACTTGGCACT	55.5	117	>chrX:154863057-154863173
		F8-6506A-mu	CCAATTATTGCTCGATACATC**G**A	53.6					

The mismatches (marked in bold and underlined) near familial variants were strategically designed according to the principle of destabilization strength in ARMS-qPCR. In most cases, these mismatches were positioned at the second-to-last nucleotide of the wild-type (WT) and mutant (MU) primers. However, to improve the specificity and differentiation between WT and MU primers, mismatches for families 2, 6, and 19 were placed at the third nucleotide from the 3' end. In addition, the primers for families 4 and 15 were designed based on their corresponding antisense sequences. This approach ensured that mismatches were properly incorporated, while maintaining similar melting temperatures (Tm) for both WT and MU primers, and also preventing any interference from secondary structures that could compromise primer performance.
